# Anatomical and Functional Outcomes with Prompt versus Delayed Initiation of Anti-VEGF in Exudative Age-Related Macular Degeneration

**DOI:** 10.3390/jcm13010111

**Published:** 2023-12-25

**Authors:** Assaf Gershoni, Edward Barayev, Rabeea H. Daood, Maureen Yogev, Orly Gal-Or, Olga Reitblat, Maria Tsessler, Michal Schaap Fogler, Raimo Tuuminen, Rita Ehrlich

**Affiliations:** 1 Ophthalmology Division, Rabin Medical Center, Petach Tikva 49414, Israel; 2 Sackler Faculty of Medicine, Tel Aviv University, Tel Aviv 69978, Israel; 3 Helsinki Retina Research Group, University of Helsinki, 00170 Helsinki, Finland; 4 Department of Ophthalmology, Kymenlaakso Central Hospital, Kotkantie 41, 48210 Kotka, Finland

**Keywords:** anti-VEGF, bevacizumab, central subfield macular thickness, exudative age-related macular degeneration, intravitreal treatment, treatment-naïve

## Abstract

Purpose: To investigate the correlation between time from diagnosis of treatment-naïve exudative age-related macular degeneration (AMD) to the introduction of anti-VEGF treatment and anatomical and functional outcomes. Design: Retrospective cohort study. Methods: Included were treatment-naïve exudative AMD patients who presented to a single tertiary medical center between 2012 and 2018. All patients were treated within the first 30 days of their diagnosis with three monthly intravitreal injections of bevacizumab. Patients were divided into three groups: group 1 (prompt anti-VEGF) were injected with bevacizumab within ten days, group 2 (intermediate anti-VEGF) within 11–20 days, and group 3 (delayed anti-VEGF) within 21–30 days from diagnosis. Baseline characteristics and clinical outcomes were compared up to two years from treatment. Results: 146 eyes of 146 patients were included. Sixty-eight patients were in the prompt anti-VEGF group, 31 in the intermediate anti-VEGF group, and 47 in the delayed anti-VEGF group. Following the induction phase of three intravitreal bevacizumab injections, the mean central subfield macular thickness (328.0 ± 115.4 µm vs. 364.6 ± 127.2 µm vs. 337.7 ± 150.1 µm, *p* = 0.432) and the best-corrected visual acuity (0.47 ± 0.38 vs. 0.59 ± 0.48 vs. 0.47 ± 0.44 logMAR units, *p* = 0.458) were comparable between the prompt, intermediate and delayed anti-VEGF groups. Anatomical and functional outcomes, treatment burden, number of relapses and eyes with second-line anti-VEGF therapy were comparable between the groups at both 1-year and 2-year timepoints. Conclusions: Our real-world evidence data emphasize that even if anti-VEGF induction cannot be initiated promptly within ten days from diagnosis of naïve exudative AMD, the visual and anatomical prognosis of the patients may not worsen if the treatment is started within one month of diagnosis.

## 1. Introduction

Age-related macular degeneration (AMD) is a major cause of blindness in Western countries, mostly when choroidal neovascularization (CNV) develops and exudative findings in the macula are present [1,2]. CNV has been shown to occur in 10% of patients with AMD, and it can lead to severe visual loss due to edema, macular hemorrhage and scarring [1,2,3,4]. The discovery that the stimulus of angiogenesis is mediated by vascular endothelial growth factors (VEGF) has revolutionized the ability to treat these patients. Nowadays, the first line of therapy and the consensus standard of care for exudative AMD consists of intravitreal anti-VEGF injections [5].

Early diagnosis and fast initiation of treatment have been shown to play a major role in reducing the extent of visual disability originating from the disease and lowering the high economic burden as a secondary effect [6,7,8,9,10]. It is well known that CNV lesions are most responsive to therapy during the first period of the disease [11]. As a direct result, even though delays can occur at any stage of treatment, they may be most damaging when they transpire between the onset of symptoms and diagnosis or between diagnosis and initial treatment. Weingessel et al. previously reported that the longer the duration between initial symptoms and intravitreal ranibizumab injections, the worse the visual outcomes [9]. Rauch et al. found that initiating treatment with ranibizumab within the first month of initial symptoms yielded better visual outcomes [12].

Bevacizumab is a recombinant humanized anti-VEGF monoclonal IgG1 antibody that binds to all isoforms of VEGF, acting to prevent angiogenesis [13]. Off-label use of bevacizumab for CNV was found to be as safe and effective as treatment with ranibizumab, and it is utilized as a first-line therapy for naïve exudative AMD in many parts of the world [14,15,16]. Despite that, there remain research gaps regarding the effect of the time frame from the primary diagnosis of exudative AMD to the first injection of bevacizumab in a real-world setting, both on anatomical and functional outcomes.

Since delays in diagnosis and initiation of treatment in healthcare systems worldwide are expected to increase, in the setting of an already over-burdened healthcare system, policy makers and ophthalmologists require information to decide whether newly diagnosed patients need to be prioritized over other patients or specific slots are to be maintained for them [11,17,18].

Here, we aimed to determine whether the time frame from the diagnosis of naïve exudative AMD to the induction of bevacizumab treatment, when administered within a month of diagnosis, is associated with the anatomical and functional outcomes in these patients.

## 2. Methods

### 2.1. Study Design

This retrospective study followed the tenets of the Declaration of Helsinki and was approved by the Institutional Review Board (IRB) of the Rabin Medical Center (Petach Tikva, Israel), Approval number 0360-21-RMC. Due to the retrospective nature of this study, written informed consent was waived by the IRB.

The study group consisted of patients who were presented to the Emergency Department diagnosed with naïve exudative AMD and who were treated with intravitreal injections of bevacizumab in a single tertiary medical center between March 2012 and February 2018.

Included were patients with a new onset of treatment naïve exudative AMD necessitating at least 3 initial monthly injections of 1.25 mg/0.05 mL bevacizumab, performed within a period of 30 days following diagnosis. Diagnosis and follow-up evaluations were performed by a retinal specialist. After the induction with 3 monthly bevacizumab injections, the patients were treated either according to the pro re nata protocol, continuing injections only if signs of exudation were present, or the treat-and-extend regimen protocol, according to the preference of the patient and the retinal specialist. All study visits included best-corrected visual acuity (BCVA) examination, a full ocular examination including dilated fundus examination, and a spectral domain optical coherence tomography (SD-OC; Spectralis OCT, Heidelberg Engineering, Heidelberg, Germany).

### 2.2. Patients

Patients with ocular comorbidities, including, e.g., corneal opacities, glaucoma, optic nerve pathologies, retinal artery or vein occlusion, and concomitant macular disorders, were excluded from the study. Previous ocular trauma, previous intravitreal injections, or past ocular surgeries other than cataract extraction were also exclusion criteria from our study.

The patients were divided into three groups as follows: group 1 (prompt anti-VEGF) included patients who were injected with bevacizumab within ten days from diagnosis (defined by the first available OCT scan representing treatment-naïve exudative AMD), group 2 (intermediate anti-VEGF) injected with bevacizumab within 11–20 days from diagnosis, and group 3 (delayed anti-VEGF) comprised of patients injected with bevacizumab within 21–30 days after diagnosis.

### 2.3. Clinical Variables

The medical charts were reviewed for the following data: patient demographics, comorbidities, ocular history, date of diagnosis, time to the first intravitreal bevacizumab injection, BCVA performed on a Snellen chart at presentation and follow-up appointments, clinical findings at dilated fundus slit-lamp examination, and central subfield macular thickness (CSMT), existence of pigment epithelial detachment (PED), intraretinal fluid (IRF), subretinal fluid (SRF) and fibrosis as recorded by SD-OCT throughout the follow-up period. Rates of patient dropout from follow-up, anti-VEGF switch to ranibizumab\aflibercept, treatment intervals and the total number of injections during the 2-year follow-up period were also recorded. Criteria for switching to other drugs included lack of anatomical improvement in OCT findings and/or deterioration in visual acuity with active disease after 3 consecutive monthly bevacizumab injections.

The main outcome measures were macular thickness (CSMT) and BCVA (LogMAR units) at 1 year. The secondary outcome measures were anti-VEGF treatment intervals and the total number of intravitreal injections, macular status, the number of relapses and the rate of anti-VEGF switch to a second-line medication (either ranibizumab or aflibercept) at 1 and 2 years.

### 2.4. Statistical Analysis

Only the first eye of each patient having treatment-naïve exudative AMD was selected for inclusion and in the case of simultaneous diagnosis, one eye was selected at random. Data were analyzed with the Statistical Package for the Social Sciences (version 27.0; SPSS, Inc., Chicago, IL, USA). For multiple group comparisons, qualitative data were analyzed with the χ² test of independence, nonparametric data for visual acuity with the Kruskall–Wallis test and continuous variables with the one-way ANOVA test. Spearman’s rho was used to evaluate the correlation between the delay of anti-VEGF administration and clinical outcomes. For statistical purposes, visual acuities were transformed to the equivalent LogMAR units. The classification for very low visual acuity was on a semi-quantitative scale, such as hand motion (HM), counting fingers (CF) and light perception (LP). The very low visual acuity measurements were converted as follows: CF 1.9, HM 2.3 and LP 2.7 LogMAR units. A *p*-value of less than 0.05 was considered statistically significant.

## 3. Results

Hundred-forty-six eyes of 146 patients were included in this study. Sixty-eight patients received their first bevacizumab injection within ten days of diagnosis, whereas 31 patients received their first bevacizumab injection between 11 and 20 days, and 47 patients received the first bevacizumab injection between 21 and 30 days from the diagnosis. In all groups, there was female predominance, and no statistically significant difference was noted between the groups in regard to comorbidities and macular or lens status (Table 1) except for the subretinal hemorrhage (*p* = 0.002, Table 1). Regarding patients’ age at baseline (*p* = 0.041, Table 1), ANOVA with Bonferroni post hoc test resulted in nonsignificant differences between prompt and delayed (*p* = 0.052) and intermediate and delayed (*p* = 0.172) anti-VEGF groups.

Baseline CSMT differed between the groups (*p* = 0.032, Table 2), but BCVA did not (*p* = 0.453, Table 2). Regarding CSMT at baseline, with the Bonferroni post hoc test, differences between intermediate and delayed anti-VEGF groups remained significant (*p* = 0.010), whereas between prompt and delayed anti-VEGF groups were nonsignificant (*p* = 0.089). Furthermore, CSMT and BCVA remained comparable between the groups after the intravitreal bevacizumab induction phase, at 1 year and at 2 years (Table 2).

**Figure 1:**
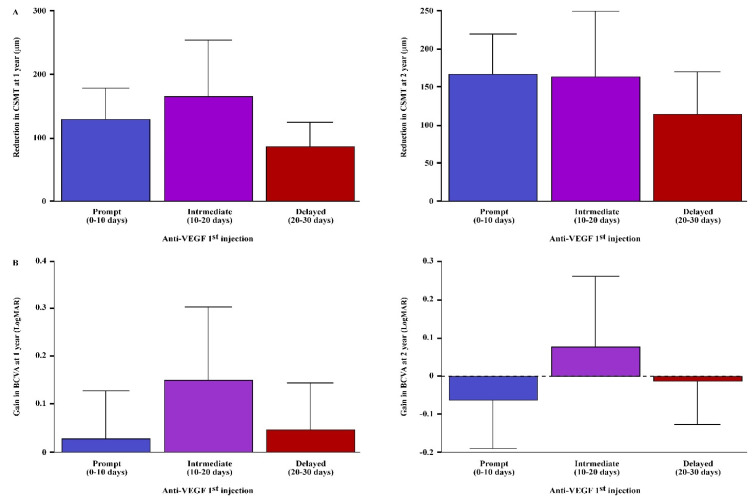
Anatomical and functional outcomes in treatment-naïve exudative AMD patients with prompt, intermediate and delayed anti-VEGF treatment. (**A**) CSMT reduction and (**B**) BCVA gain at 1 year and 2 years in prompt, intermediate and delayed anti-VEGF treatment groups in treatment-naïve exudative AMD patients. BCVA; best-corrected visual acuity, CSMT; central subfield macular thickness.

Macular status and disease activity (PED, IRF, and SRF) did not differ between the study groups at any timepoint (Table 3). No correlations were found between the time from exudative AMD diagnosis to the initiation of bevacizumab therapy in regard to CSMT and BCVA at any follow-up timepoint (Table 4, Figure 1 and Figure S1). Moreover, even after adjusting for anti-VEGF treatment protocol as a covariate, correlations between the time from exudative AMD diagnosis to the initiation of bevacizumab therapy in regard to CSMT and BCVA remained nonsignificant after intravitreal bevacizumab induction phase (R = 0.019, *p* = 0.840 and R = 0.030, *p* = 0.767, Table 4, respectively), at 1 year (R = 0.071, *p* = 0.425 and R = 0.005, *p* = 0.997, Table 4, respectively) and at 2 years (R = 0.069, *p* = 0.426 and R = 0.034, *p* = 0.643, Table 4, respectively).

Eighty-nine percent of the cohort had complete data up to two years from presentation. There was no difference between the groups regarding the percentage of patients that did not reach the 2-year follow-up (*p* = 0.785, Table 5). The time from wAMD diagnosis to the initiation of bevacizumab treatment did not affect the anti-VEGF switch rates to ranibizumab/aflibercept at the 1- and 2-year timepoints (*p* = 0.358 and *p* = 0.961, respectively, Table 5). Moreover, the time from wAMD diagnosis to the initiation of bevacizumab treatment did not influence the treatment interval, which was 6.85 ± 1.19 vs. 6.80 ± 1.26 vs. 6.78 ± 1.21 weeks in the prompt, intermediate and delayed anti-VEGF groups, respectively, at the 1 year (*p* = 0.978, Table 5), and 7.52 ± 1.50 vs. 7.73 ± 1.94 vs. 7.04 ± 2.23 weeks, respectively, at the 2-year timepoints (*p* = 0.462, Table 5). The cumulative number of injections was comparable between the study groups at the 1- and 2-year timepoints (*p* = 0.928 and *p* = 0.209, respectively, Table 5).

## 4. Discussion

In this real-world study, we found no correlation between the timing of the first administered bevacizumab injection, if given within the first month after diagnosis of exudative AMD, and the anatomical and clinical outcomes during two years of follow-up. The patients in our study showed improvement following the initiation of treatment regardless of whether they were being treated within a week or a month from diagnosis. Previous studies suggest that prompt treatment is advised, and our data complement these assumptions, showing that delaying treatment past the first week from diagnosis did not result in worse functional or anatomical outcomes [9,12].

Studies by Klein et al. and Vander et al. estimated that untreated CNV in AMD progresses at a mean rate of about 9–10 µm per day [19,20]. These findings, along with the aforementioned studies, stress the importance of early diagnosis and treatment in order to achieve maximal improvement in visual outcome, as well as reduced disability rates in patients with exudative AMD.

Delays in the initiation of treatment have been well documented in previous studies, including delays in the first presentation to a healthcare provider, referral from primary care and the initiation of secondary care treatment [6,11,12,21]. In addition, the COVID-19 pandemic also had a detrimental effect on the timing of treatment induction, as there was a 48.5–91.7% drop in the number of intravitreal injections compared to the time before the pandemic [22,23,24]. Another possible reason for this delay of treatment may be the enrollment process for multicenter prospective studies. Although long delays between the appearance of initial symptoms and initiation of therapy have been shown to lower the chances of improvement after treatment [21], apart from educating the population to note the symptoms and approach their doctor for any change observed, there is no way to control the delay prior to the first ophthalmologic evaluation. Therefore, it is important to analyze the significance of the elapsed time from diagnosis to initiation of treatment, as we may tailor our management accordingly. In their study from 2005, Olivier-Fernandez et al. found that this time frame was associated with the progression of visual loss, but the median time frame in their study was 28 days, which nowadays is considered quite excessive [18]. Takahashi et al. had a larger variation in treatment delay, up to 105 days, and found a connection between the delay in initiation of treatment and worse outcomes in terms of visual acuity [17] Lim et al. also observed the effects of a longer delay in treatment initiation than our study, comparing a delay of 21 weeks or more to 7 weeks or less, and found a statistically significant difference in favor of prompt treatment [21]. The Royal College of Ophthalmologists suggests that new patients with AMD should not have to wait more than one week from referral to clinic and not more than one week from clinic to treatment if needed [25].

Here, we show that no correlation was found between the timing of anti-VEGF treatment when initiated within the month from diagnosis and anatomical and visual outcomes. Macular thickness, disease activity and BCVA did not differ between the prompt, intermediate and delayed anti-VEGF groups. Furthermore, at the 2-year timepoint, the mean BCVA returned to the baseline in all groups. These data are in accordance with other studies showing real-life results with bevacizumab for exudative AMD [26]. The explanation of these results might be that the time from initiation of the disease to diagnosis in the real world could be long, and after a certain point, there may be no difference in response when treatment is initiated within one month. Longer delays, such as in the aforementioned studies, could be more significant in terms of visual outcomes. The definitions of time from diagnosis to treatment might differ between studies and populations. It is possible that the period from the first time a patient is examined by a physician, the images are performed, the patient is referred to treatment, and the treatment is initiated is already too long. We defined the time from diagnosis to treatment as the time from the first OCT study showing exudative AMD to the first anti-VEGF injection. Regular screening of the population, especially individuals that have increased risk, might improve this outcome, and with early diagnosis, early treatment may show more beneficial results. Nevertheless, it seems that other morphological and demographic prognostic indicators are also influential to exudative AMD treatment response and outcomes more so than the few days’ change in the time to initiation of bevacizumab therapy [27].

We speculate that the reasons for the lack of difference in treatment response might be multifactorial, as each individual is different; while some cases are not affected by the delay in treatment, in others, bleeding could form on the next day from examination if anti-VEGF is not initiated. Other reasons for our results could be the chronicity of disease and scarring in some patients. Also, different individuals respond differently to the same treatment. For example, previous studies exploring the inflammatory response to bevacizumab found an increase in inflammation and even severe ocular inflammation, while others found a reduction in inflammatory response following bevacizumab administration in a uveitis model [28,29,30,31].

It is important to emphasize that in Israel, we have statutory public health insurance for every citizen, and when an indication for a bevacizumab injection arises in a patient with exudative AMD, his insurance will cover its cost. However, like other healthcare systems throughout the world, our system is immensely burdened by the magnitude of demand for anti-VEGF therapy, which creates pressure for efficient and timely provision of services. Budgetary constraints, as well as procedural obstacles and long waiting times, may defer the initiation of treatment. We found that a delay in anti-VEGF induction of up to one month among treatment-naïve exudative AMD patients does not increase the treatment burden as it neither affects the treatment interval nor the number of injections at 1-year and 2-year endpoints. In addition, there is no difference between the groups in the need to switch to second-line anti-VEGF drugs, which also constitutes great expenditures on the already limited public healthcare resources.

The main limitations of our study are its retrospective nature and medium sample size. In addition, the nonrandomized study setting may have led to a possible allocation bias between the study groups, as patients with a more severe disease may have been referred for more prompt treatment. The visual acuity at the start of treatment in our study was slightly worse compared to other studies, which could partially explain the less favorable results in terms of posttreatment BCVA. However, this represents the population encountered in our clinic and in our country, which might suggest that patients come to obtain treatment quite late, as 80% of them already had IRF and 10% had fibrosis at the start of their treatment. In addition, although the differences in the initial CSMT between intermediate and delayed may have introduced a bias, no statistically significant differences were shown posttreatment in regard to CSMT and BCVA. Another limitation stems from the fact we do not have data regarding the time interval from the onset of symptoms to diagnosis. However, although this information may have been useful in our analysis, it does not reduce the significance of our results, as it represents real-life circumstances. Elderly patients with AMD who seek treatment often cannot point the exact time their symptoms first arose. Also, although we found no anatomical or functional differences between the study groups, since the disease course of exudative AMD may vary from person to person, our results may not be applicable to all cases. While initial improvement in mean BCVA and mean CMT was demonstrated in all groups with no statistically significant differences between any of them, we do not know if our results would have followed had we known the exact time from the initial CNV occurrence to treatment. Finally, the objective outcome measurements of our study were CSMT and BCVA, as these are often used to evaluate treatment response. More specific anatomic changes, including IRF, SRF, PED and specific retinal layers integrity and their correlation to the timing of treatment initiation, should be evaluated in future studies.

In conclusion, our real-world evidence data suggest that even if treatment with bevacizumab cannot be initiated promptly within the first 10 days after diagnosis of naïve exudative AMD, the visual and anatomical prognosis of the patients and the treatment burden does not worsen if bevacizumab is initiated within a month of diagnosis.

## Figures and Tables

**Table 1:** Baseline variables.

	Prompt Anti-VEGF N = 68	Intermediate Anti-VEGF N = 31	Delayed Anti-VEGF N = 47	*p* =
Age (years) at diagnosis	81.1 ± 7.3	81.0 ± 7.7	77.5 ± 8.8	0.041
Male:Female (*n*/%)	28:40 (41:59%)	15:16 (48:52%)	19:28 (43:57%)	0.752
Laterality (right:left)	32:36 (47:53%)	16:15 (52:48%)	25:22 (53:47%)	0.795
Lens status (phakic:pseudophakic)	23:45 (34:66%)	13:18 (42:58%)	19:28 (40:60%)	0.561
**Comorbidities**				
Diabetes	15 (22%)	8 (26%)	11 (23%)	0.916
Glaucoma	6 (9%)	4 (13%)	5 (11%)	0.845
Hypertension	49 (72%)	22 (71%)	29 (62%)	0.441
Ischemic heart disease	17 (25%)	9 (29%)	8 (17%)	0.438
**Macular status**				
Fibrosis	10%	3%	15%	0.197
IRF	74%	71%	64%	0.369
PED	40%	42%	43%	0.984
SRF	91%	87%	75%	0.366
Subretinal hemorrhage	47%	39%	15%	0.002
**CNV type**				
Type 1	96%	97%	96%	0.960
Type 2	4%	3%	4%	
**Treatment regimen**				
PRN	60%	52%	62%	0.821
T&E	40%	48%	38%	

Data are given as mean ± SD or absolute numbers with proportions. For multiple group comparisons, qualitative data were analyzed with the χ² test of independence and continuous variables with the one-way ANOVA test. IRF; intraretinal fluid, PED; pigment epithelial detachment, PRN; pro re nata (as needed), SRF; subretinal fluid, T&E; treat-and-extend.

**Table 2:** Macular thickness and visual acuity.

	Prompt Anti-VEGF N = 68	Intermediate Anti-VEGF N = 31	Delayed Anti-VEGF N = 47	*p* =
**CSMT (µm)**				
Pre	479.7 ± 163.3	527.3 ± 214.0	424.1 ± 149.0	0.032
After anti-VEGF induction phase *	328.0 ± 115.4	364.6 ± 127.2	337.7 ± 150.1	0.432
At 1 year	339.4 ± 163.9	351.2 ± 120.8	335.3 ± 124.1	0.898
At 2 years	335.6 ± 117.6	348.6 ± 138.3	323.9 ± 124.3	0.732
**BCVA (LogMAR)**				
Pre	0.59 ± 0.45	0.75 ± 0.57	0.59 ± 0.50	0.453
After anti-VEGF induction phase *	0.47 ± 0.38	0.59 ± 0.48	0.47 ± 0.44	0.458
At 1 year	0.54 ± 0.51	0.62 ± 0.44	0.52 ± 0.47	0.449
At 2 years	0.63 ± 0.54	0.65 ± 0.54	0.54 ± 0.46	0.536

Data are given as mean ± SD. For multiple group comparisons, nonparametric data for visual acuity were analyzed with the Kruskall–Wallis test, and continuous variables for macular thickness with the one-way ANOVA test. For statistical purposes, visual acuities were transformed to the equivalent LogMAR units. The classification for very low visual acuity was on a semi-quantitative scale, such as hand motion (HM), counting fingers (CF) and light perception (LP). The very low visual acuity measurements were converted as follows: CF 1.9, HM 2.3 and LP 2.7 LogMAR units. BCVA; best-corrected visual acuity, CSMT; central subfield macular thickness, LogMAR; logarithm of the minimum angle of resolution. * Induction of three monthly bevacizumab injections.

**Table 3:** Wet age-related macular degeneration activity.

	Prompt Anti-VEGF N = 68	Intermediate Anti-VEGF N = 31	Delayed Anti-VEGF N = 47	*p* =
**Macular status after induction**				
IRF	41%	39%	38%	0.914
PED	37%	45%	28%	0.439
SRF	46%	48%	47%	0.669
**Macular status at 1 year**				
IRF	22%	26%	26%	0.936
PED	29%	36%	45%	0.380
SRF	25%	29%	28%	0.941
**Macular status at 2 years**				
IRF	31%	39%	28%	0.707
PED	41%	45%	49%	0.680
SRF	28%	39%	26%	0.688

Data are given as mean ± SD or absolute numbers with proportions. For multiple groups comparisons, qualitative data were analyzed with the χ² test of independence. IRF; intraretinal fluid, PED; pigment epithelial detachment, SRF; subretinal fluid.

**Table 4:** Correlations between time from wAMD diagnosis to initiation of anti-VEGF therapy and anatomical and functional parameters.

Time from wAMD Diagnosis (days)		
	R =	*p* =
**CSMT (µm)**		
After anti-VEGF induction *	0.007	0.934
Δ from baseline	0.160	0.054
At 1 year	0.070	0.428
Δ from baseline	0.052	0.558
At 2 years	0.064	0.498
Δ from baseline	0.073	0.436
**BCVA (LogMAR)**		
After anti-VEGF induction *	0.017	0.836
Δ from baseline	0.101	0.227
At 1 year	0.004	0.965
Δ from baseline	0.051	0.553
At 2 years	0.030	0.741
Δ from baseline	0.014 (4.3%)	0.877

BCVA; best-corrected visual acuity, CSMT; central subfield macular thickness, logMAR; logarithm of the minimum angle of resolution. Δ = change. * Induction of three monthly bevacizumab injections.

**Table 5:** Patients’ dropout and treatment burden.

	Prompt Anti-VEGF N = 68	Intermediate Anti-VEGF N = 31	Delayed Anti-VEGF N = 47	*p* =
**Dropout during follow-up (N/%)**	6 (9%)	3 (10%)	6 (13%)	0.785
**Anti-VEGF switch (ranibizumab/aflibercept)**				
At 1 year	14%	7%	19%	0.358
At 2 years	23%	25%	24%	0.961
Anti-VEGF treatment interval (weeks) *				
At 1 year	6.85 ± 1.19	6.80 ± 1.26	6.78 ± 1.21	0.978
At 2 years	7.52 ± 1.50	7.73 ± 1.94	7.04 ± 2.23	0.462
**Cumulative number of anti-VEGF injections**				
At 1st year (after induction phase)	5.03 ± 1.77	5.19 ± 1.59	5.08 ± 1.67	0.928
At 2nd year	5.88 ± 2.77	4.68 ± 2.58	5.53 ± 2.79	0.209

Data are given as mean ± SD or absolute numbers with proportions. For multiple group comparisons, qualitative data were analyzed with the χ² test of independence and continuous variables with the one-way ANOVA test. * Treatment interval between the study groups was compared among a subgroup of patients who were anti-VEGF treated according to the treat-and-extend regimen protocol (N = 60 patients in total).

## Data Availability

All data relevant to the study are included in the article or uploaded as supplementary information.

## Supplementary Materials

The following supporting information can be downloaded at: https://www.mdpi.com/article/10.3390/jcm13010111/s1, Figure S1: Scatterplot of Anatomical and functional outcomes in treatment-naïve exudative AMD patients after anti-VEGF treatment. (A) CSMT reduction and (B) BCVA gain at 1 year after anti-VEGF treatment in treatment-naïve exudative AMD patients, shown in correlation to time from diagnosis to first anti-VEGF injection. BCVA; best-corrected visual acuity, CSMT; central subfield macular thickness.

## References

[1] Lim L.S., Mitchell P., Seddon J.M., Holz F.G., Wong T.Y. (2012). Age-Related Macular Degeneration. Lancet.

[2] Klaver C.C.W., Wolfs R.C.W., Vingerling J.R., Hofman A., De Jong P.T.V.M. (1998). Age-Specific Prevalence and Causes of Blindness and Visual Impairment in an Older Population: The Rotterdam Study. Arch. Ophthalmol..

[3] Ambati J., Ambati B.K., Yoo S.H., Ianchulev S., Adamis A.P. (2003). Age-Related Macular Degeneration: Etiology, Pathogenesis, and Therapeutic Strategies. Surv. Ophthalmol..

[4] Burgess D.B., Hawkins B.S., Jefferys J.L., Bressler N.M., Bressler S.B., Hiner C.J., Javornik N.B., Orth D.H., Wilkinson C.P. (1993). MPSG Five-Year Follow-up of Fellow Eyes of Patients with Age-Related Macular Degeneration and Unilateral Extrafoveal Choroidal Neovascularization. Arch. Ophthalmol..

[5] Barouch F.C., Miller J.W. (2004). Anti-Vascular Endothelial Growth Factor Strategies for the Treatment of Choroidal Neovascularization from Age-Related Macular Degeneration. Int. Ophthalmol. Clin..

[6] Arias L., Armadá F., Donate J., García-Arumí J., Giralt J., Pazos B., Pĩero A., Martínez F., Mondéjar J.J., Ortega I. (2009). Delay in Treating Age-Related Macular Degeneration in Spain Is Associated with Progressive Vision Loss. Eye.

[7] Bonastre J., Le Pen C., Soubrane G., Quentel G. (2003). The Burden of Age-Related Macular Degeneration: Results of a Cohort Study in Two French Referral Centres. Pharmacoeconomics.

[8] Lotery A., Xu X., Zlatava G., Loftus J. (2007). Burden of Illness, Visual Impairment and Health Resource Utilisation of Patients with Neovascular Age-Related Macular Degeneration: Results from the UK Cohort of a Five-Country Cross-Sectional Study. Br. J. Ophthalmol..

[9] Weingessel B., Hintermayer G., MacA S.M., Rauch R., Vecsei-Marlovits P.V. (2012). The Significance of Early Treatment of Exudative Age-Related Macular Degeneration: 12 Months’ Results. Wien. Klin. Wochenschr..

[10] Muether P.S., Hermann M.M., Koch K., Fauser S. (2011). Delay between Medical Indication to Anti-VEGF Treatment in Age-Related Macular Degeneration Can Result in a Loss of Visual Acuity. Graefes Arch. Clin. Exp. Ophthalmol..

[11] Sim P.Y., Gajree S., Dhillon B., Borooah S. (2017). Investigation of Time to First Presentation and Extrahospital Factors in the Treatment of Neovascular Age-Related Macular Degeneration: A Retrospective Cross-Sectional Study. BMJ Open.

[12] Rauch R., Weingessel B., MacA S.M., Vecsei-Marlovits P.V. (2012). Time to First Treatment: The Significance of Early Treatment of Exudative Age-Related Macular Degeneration. Retina.

[13] Algvere P.V., Steén B., Seregard S., Kvanta A. (2008). A Prospective Study on Intravitreal Bevacizumab (Avastin) for Neovascular Age-Related Macular Degeneration of Different Durations. Acta Ophthalmol..

[14] Solomon S.D., Lindsley K.B., Krzystolik M.G., Vedula S.S., Hawkins B.S. (2016). Intravitreal Bevacizumab Versus Ranibizumab for Treatment of Neovascular Age-Related Macular Degeneration: Findings from a Cochrane Systematic Review. Ophthalmology.

[15] Berg K., Hadzalic E., Gjertsen I., Forsaa V., Berger L.H., Kinge B., Henschien H., Fossen K., Markovic S., Pedersen T.R. (2016). Ranibizumab or Bevacizumab for Neovascular Age-Related Macular Degeneration According to the Lucentis Compared to Avastin Study Treat-and-Extend Protocol. Ophthalmology.

[16] DF M., MG M., GS Y., JE G., SL F., GJ J. (2011). Ranibizumab and Bevacizumab for Neovascular Age-Related Macular Degeneration. N. Engl. J. Med..

[17] Takahashi H., Ohkubo Y., Sato A., Takezawa M., Fujino Y., Yanagi Y., Kawashima H. (2015). Relationship between Visual Prognosis and Delay of Intravitreal Injection of Ranibizumab When Treating Age-Related Macular Degeneration. Retina.

[18] Oliver-Fernandez A., Bakal J., Segal S., Shah G.K., Dugar A., Sharma S. (2005). Progression of Visual Loss and Time between Initial Assessment and Treatment of Wet Age-Related Macular Degeneration. Can. J. Ophthalmol..

[19] Klein M.L., Jorizzo P.A., Watzke R.C. (1989). Growth Features of Choroidal Neovascular Membranes in Age-Related Macular Degeneration. Ophthalmology.

[20] Vander J.F., Morgan C.M., Schatz H. (1989). Growth Rate of Subretinal Neovascularization in Age-Related Macular Degeneration. Ophthalmology.

[21] Lim J.H., Wickremasinghe S.S., Xie J., Chauhan D.S., Baird P.N., Robman L.D., Hageman G., Guymer R.H. (2012). Delay to Treatment and Visual Outcomes in Patients Treated with Anti-Vascular Endothelial Growth Factor for Age-Related Macular Degeneration. Am. J. Ophthalmol..

[22] Wasser L.M., Weill Y., Brosh K., Magal I., Potter M., Strassman I., Gelman E., Koslowsky M., Zadok D., Hanhart J. (2020). The Impact of COVID-19 on Intravitreal Injection Compliance. SN Compr. Clin. Med..

[23] dell’Omo R., Filippelli M., Virgili G., Bandello F., Querques G., Lanzetta P., Avitabile T., Viola F., Reibaldi M., Semeraro F. (2022). Effect of COVID-19-Related Lockdown on Ophthalmic Practice in Italy: A Report from 39 Institutional Centers. Eur. J. Ophthalmol..

[24] Carnevali A., Giannaccare G., Gatti V., Scuteri G., Randazzo G., Scorcia V. (2021). Intravitreal Injections during COVID-19 Outbreak: Real-World Experience from an Italian Tertiary Referral Center. Eur. J. Ophthalmol..

[25] Chakravarthy U., Williams M., Amoaku W., Bailey C., Bishop P., Brand C., Chong V., Downes S., Evans J., Lotery A. (2013). The Royal College of Ophthalmologists Guidelines on AMD: Executive Summary. Eye.

[26] Beykin G., Grunin M., Averbukh E., Banin E., Hemo Y., Chowers I. (2015). Bevacizumab Treatment for Neovascular Age-Related Macular Degeneration in the Setting of a Clinic: “Real Life” Long-Term Outcome. BMC Ophthalmol..

[27] Ashraf M., Souka A., Adelman R.A. (2018). Age-Related Macular Degeneration: Using Morphological Predictors to Modify Current Treatment Protocols. Acta Ophthalmol..

[28] Raja M.S., Goldsmith C., Burton B.J.L. (2010). Intraocular Inflammation with Intravitreal Bevacizumab (Avastin). Br. J. Ophthalmol..

[29] Georgopoulos M., Polak K., Prager F., Prünte C., Schmidt-Erfurth U. (2009). Characteristics of Severe Intraocular Inflammation Following Intravitreal Injection of Bevacizumab (Avastin). Br. J. Ophthalmol..

[30] Mérida S., Sancho-Tello M., Almansa I., Desco C., Peris C., Moreno M.L., Villar V.M., Navea A., Bosch-Morell F. (2018). Bevacizumab Diminishes Inflammation in an Acute Endotoxin-Induced Uveitis Model. Front. Pharmacol..

[31] EL-Hajjar L., Jalaleddine N., Shaito A., Zibara K., Kazan J.M., El-Saghir J., El-Sabban M. (2019). Bevacizumab Induces Inflammation in MDA-MB-231 Breast Cancer Cell Line and in a Mouse Model. Cell Signal..

