# Supplementary figures and images for: Anatomical and Functional Outcomes with Prompt versus Delayed Initiation of Anti-VEGF in Exudative Age-Related Macular Degeneration
Source: J Clin Med. 2023 Dec 25;13(1):111. doi: 10.3390/jcm13010111 (PMC10779608; doi:10.3390/jcm13010111)

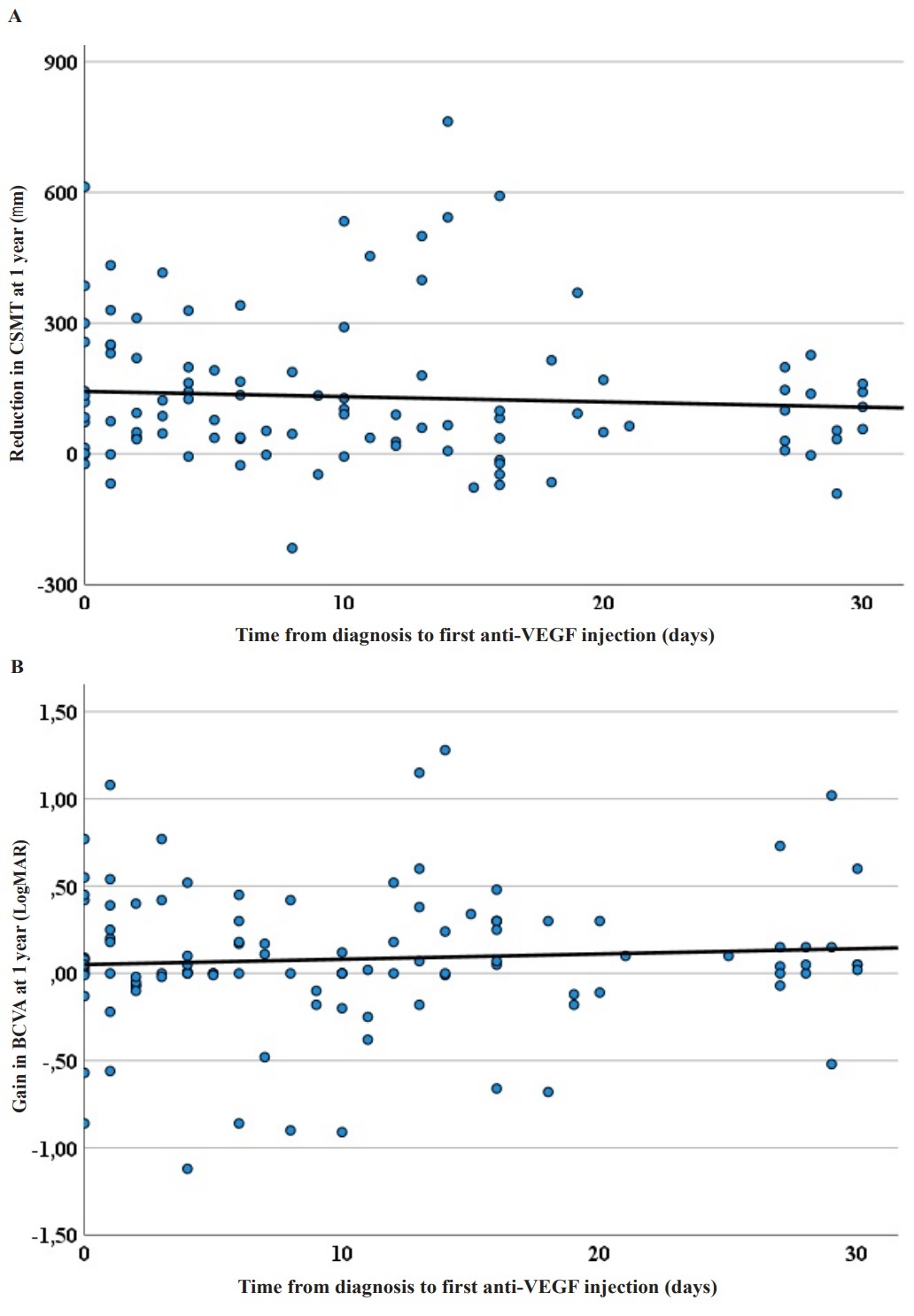

Supplement: Supplementary file 1 [file jcm-13-00111-s001.zip › jcm-2649196-supplementary.jpg]
